# Dipeptidyl peptidase 3 and thimet oligopeptidase 1 knockdown support tumor-specific immune responses to whole cell cancer vaccines and tumor cell death in vivo

**DOI:** 10.1186/2051-1426-1-S1-P214

**Published:** 2013-11-07

**Authors:** Jaba Gamrekelashvili, Miaojun Han, Tamar Kapanadze, Josef Wissing, Chi Ma, Lothar Jaensch, Todd Armstrong, Liz Jaffee, Ayla White, Deborah Citrin, Firouzeh Korangy, Tim Greten

**Affiliations:** 1 Center for Cancer Research, NCI, Bethesda, MD, USA; 2 Johns Hokpins University, Baltimore, MD, USA; 3 HZI, Braunschweig, Germany

## Background and aims

Tumor cell vaccines are widely used as a potential therapeutic. Different approaches are currently being applied to kill tumor cell vaccines prior to injection to prevent outgrowth of metastasis at the site of injection. We and others have previously shown that the mode by which tumor cells die can have significant effects on anti-tumor specific immune responses. Here, we have studied how tumor cell necrosis impairs immune responses and describe a new strategy to enhance tumor-specific immune responses to dying tumor cells.

## Methods

B78HI wt, B78HI-Ova, CT26, CT26-Ova, EG7 and NT2.5 tumor cells and C57/BL/6, OT-I and FVB/N mice were used. Tumor cell death was induced by 3 freeze-thaw cycles in vitro and gamma-irradiation in vivo and in vitro. T cell responses were analyzed by tetramer staining, in vivo CTL and intracellular cytokine analysis. Growth of subcutaneous tumors was monitored 3 times a week. Gene knockdowns were done by transfection of tumor cells with corresponding shRNA constructs. Conventional gel chromatography followed by mass spectrometry was performed to identify genes of interest.

## Results

Freeze thawed (necrotic) tumor cells failed to induce anti-tumor immune responses. In contrast heating of necrotic tumor cells prior to injection or loading heated necrotic lysates onto dendritic cells reversed their non-immunogenicity and led to the induction of a potent anti-tumor immune response determined by in vivo CTL, tetramer staining and IFN-gamma response. Similar results were observed in a vaccination setting suggesting that necrotic cells contain a factor inhibiting induction of T cell responses. In order to identify this factor we set up an in vitro system to evaluate the effect of tumor necrosis on cross-priming. We identified two proteins, dipeptidyl peptidase 3 (DPP3) and thimet oligopeptidase 1 (TOP1) in necrotic cells, which were able to block the induction of T cell responses to dying tumor cells in vitro and in vivo. In contrast knockdown of DPP3 and TOP1 reversed the non-immunogenicity of necrotic tumor cells and supported tumor-specific T cell responses to dying tumor cells in vitro and in vivo in different tumor models. Finally, TOP1 knockdown increased tumor-specific immune responses to subcutaneous tumors after radiation induced tumor cell death.

## Conclusion

DPP3 and TOP1 prevent tumor-specific immune responses to dying tumor cells and represent important targets to improve anti-tumor immunity to dying tumor cells.

